# Protection against dengue disease by synthetic nucleic acid antibody prophylaxis/immunotherapy

**DOI:** 10.1038/srep12616

**Published:** 2015-07-29

**Authors:** Seleeke Flingai, Emily M. Plummer, Ami Patel, Sujan Shresta, Janess M. Mendoza, Kate E. Broderick, Niranjan Y. Sardesai, Kar Muthumani, David B. Weiner

**Affiliations:** 1 Department of Pathology and Laboratory Medicine, Perelman School of Medicine at the University of Pennsylvania, Philadelphia, PA 19104, USA; 2 Division of Vaccine Discovery, La Jolla Institute for Allergy and Immunology, 9420 Athena Circle, La Jolla, CA 92037, USA; 3 Inovio Pharmaceuticals Inc., Plymouth Meeting, PA 19462, USA

## Abstract

Dengue virus (DENV) is the most important mosquito-borne viral infection in humans. In recent years, the number of cases and outbreaks has dramatically increased worldwide. While vaccines are being developed, none are currently available that provide balanced protection against all DENV serotypes. Advances in human antibody isolation have uncovered DENV neutralizing antibodies (nAbs) that are capable of preventing infection from multiple serotypes. Yet delivering monoclonal antibodies using conventional methods is impractical due to high costs. Engineering novel methods of delivering monoclonal antibodies could tip the scale in the fight against DENV. Here we demonstrate that simple intramuscular delivery by electroporation of synthetic DNA plasmids engineered to express modified human nAbs against multiple DENV serotypes confers protection against DENV disease and prevents antibody-dependent enhancement (ADE) of disease in mice. This synthetic nucleic acid antibody prophylaxis/immunotherapy approach may have important applications in the fight against infectious disease.

Nearly 400 million dengue infections occur each year^1^, and cases of dengue fever (DF) and the potentially fatal dengue hemorrhagic fever/dengue shock syndrome (DHF/DSS) have grown in recent decades. The geographical reach of dengue has expanded to include over 100 countries, resulting in a significant health and economic burden worldwide^1^,^2^. While primary DENV infection is thought to elicit persistent and effective immunity against reinfection with the same serotype, only short-term protection is elicited against other DENV serotypes^3^. Disease severity is associated with subsequent heterotypic infection, during which non- or sub-neutralizing levels of cross-reactive antibodies from prior infection form immune complexes with DENV that lead to increased infection of Fcγ receptor (FcγR)-bearing monocytes and macrophages^4^,^5^,^6^. This phenomenon, known as antibody-dependent enhancement (ADE), gives rise to one of the greatest challenges in developing a dengue vaccine: eliciting balanced, neutralizing immunity across multiple serotypes while minimizing the risk of ADE. A recent live-attenuated, quadrivalent vaccine candidate from Sanofi has shown promising protective efficacy against DENV1, 3, and 4, but underwhelming protection against DENV2^7^,^8^,^9^, a serotype frequently associated with severe disease from secondary infections^10^. Furthermore, whether vaccine-induced humoral responses can overcome the threat of ADE in vaccinees over time remains to be seen.

Passive immunization studies have shown that neutralizing monoclonal or polyclonal antibodies can provide cross-serotype protection against DENV infection in mice^11^,^12^,^13^,^14^,^15^,^16^ and non-human primates (NHPs)^12^. Yet monoclonal antibody delivery in humans is incredibly expensive, creating cost-prohibitive barriers for most regions of the world where such therapy would be needed. Developing new methods for delivering cross-reactive, neutralizing monoclonal antibodies into the circulation may provide rapid, complete protection against DENV-associated disease.

One such approach involves vector-mediated gene transfer of monoclonal antibodies. Several studies have demonstrated the effectiveness of this delivery strategy in protecting NHPs against SIV^17^, humanized mice against HIV^18^,^19^, and mice and ferrets against influenza^20^,^21^,^22^. While these studies have employed intramuscular or intranasal administration of adeno-associated virus (AAV) vectors to produce protective antibodies, our interest in DNA plasmids has led us to explore whether such vectors can be used to deliver neutralizing monoclonal antibodies into the circulation. DNA plasmids represent an interesting vector model for gene transfer: they have an excellent safety profile, and unlike viral vectors, have no vector-associated serology, allowing for repeat delivery^23^,^24^,^25^. As a proof of concept, we previously constructed optimized DNA plasmids capable of expressing Fab fragments of the HIV-1 broadly neutralizing antibody VRC01 in mice after intramuscular injection and *in vivo* electroporation (EP), resulting in mouse sera that neutralized multiple strains of HIV-1^26^. To date, however, no vector system has been used to deliver neutralizing, protective anti-DENV IgG antibodies into any animal model.

Here, we describe an approach to delivering cross-reactive neutralizing antibodies against DENV into the circulation using DNA plasmid-mediated antibody gene transfer. This synthetic DNA-encoded antibody approach (DMAb) produces biologically relevant levels of mAbs after a single intramuscular injection of antibody-encoding DNA. As this approach allows for genetic tailoring of the exact features of the desired antibody, we further studied the role of Fc region modifications on protection. We demonstrate that intramuscular delivery of a DNA plasmid encoding an anti-DENV human IgG1 nAb, with an Fc region mutation that abrogates FcγR binding, protects mice from both virus-only infection and antibody-enhanced lethal infection.

## Results

### DMAb optimization and *in vitro* characterization

**Figure 1:**
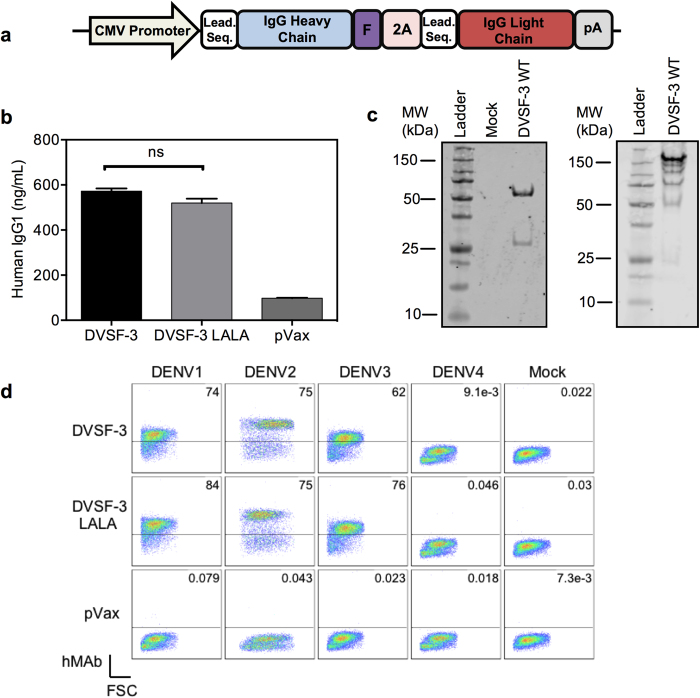
*In vitro* expression of human anti-DENV neutralizing mAbs by DMAb. (**a**) Schematic illustration of DNA plasmid used for DMAb; antibody heavy and light chain sequences are separated by a combination of furin and 2A cleavage sites. (**b**) ELISA quantification analysis of human IgG in supernatants of pDVSF-3 WT- or LALA-transfected 293T cells. The data displayed are the mean of triplicate values +/− standard error of the mean (SEM) and are representative of three independent experiments. (**c**) Western blot analysis of pDVSF-3 WT-transfected 293T supernatants containing DVSF-3 WT. Antibodies were purified by Protein A spin columns and separated by SDS-PAGE under reducing (left) and non-reducing (right) conditions.(**d**) Vero cells were either uninfected (Mock) or infected by DENV1, 2, 3, or 4, then fixed, permeabilized, and stained with supernatants of pDVSF-3 WT- or LALA-transfected 293T cells. The data displayed are representative of two independent experiments.

The expression of human IgG antibodies from DNA-based vectors has briefly been explored in the past^27^ and resulted in low levels of serum-detectable antibodies *in vivo*. However, subsequent genetic optimizations to DNA plasmids and accompanying delivery systems, particularly EP, have resulted in increased expression of desired proteins^28^. With this in mind, we systematically optimized DMAb DNA through the creation of multiple iterations of two- and single-plasmid antibody-encoding DNA cassettes, with the aim of increasing human IgG production from DMAb *in vivo* (Supplementary Fig. 1). Dosage studies of two-plasmid DMAb delivery in C57BL/6 mice showed a dose-dependent increase in serum-detectable human IgG levels, and a comparison between two- and single-plasmid DMAb constructs revealed a slight increase in expression from the single-plasmid condition (Supplementary Fig. 1). Ultimately, we designed and constructed highly optimized DNA plasmids encoding the heavy and light chains of the anti-DENV antibody DV87.1, a human IgG1 mAb that has been well characterized for its ability to neutralize DENV1–3^14^. Two optimized plasmids were constructed: pDVSF-3 WT, which encodes for the heavy and light chains of DV87.1, and pDVSF-3 LALA, which encodes for an Fc region-modified version of DV87.1 with abrogated FcγR binding by way of two leucine-to-alanine (LALA) mutations in the CH2 region^29^ that have been shown to eliminate antibody-dependent enhancement^14^. In order to express a full-length antibody from a single open reading frame, the heavy and light chain genes were separated by a furin cleavage site and a P2A self-processing peptide. Each transgene was genetically optimized, synthesized, and subcloned into a modified pVax1 mammalian expression vector (Fig. 1a). The plasmids were transfected into human embryonic kidney (HEK) 293T cells, and secreted antibody levels in the supernatant were quantified after 48 hours by enzyme-linked immunosorbent assay (ELISA) (Fig. 1b). Both pDVSF-3 WT and pDVSF-3 LALA resulted in 600 ng/mL of human IgG, confirming that the plasmids can express human IgG, and that the LALA mutation has no effect on antibody expression levels *in vitro*. To confirm proper antibody assembly, DVSF-3 and DVSF-3 LALA antibodies were collected from supernatants of transfected HEK293T cells and separated by SDS-PAGE gel for Western blot analysis (Fig. 1c). The heavy and light chain proteins were at their expected molecular weights, suggesting proper protein cleavage and antibody assembly.

To assess the biological activity of the antibodies, we first performed a binding ELISA assay that measures whether the antibody-containing supernatant can bind to recombinant DENV1-3 E proteins. The supernatants of HEK293T cells that secreted either DVSF-3 WT or DVSF-3 LALA antibodies were able to recognize DENV1–3 E proteins, while DENV4 went unrecognized, as expected (Supplementary Fig. 2). Additionally, DVSF-3 WT- and DVSF-3 LALA-containing supernatants were able to stain Vero cells infected with DENV1–3, whereas Vero cells infected with DENV4 were not stained by the supernatants (Fig. 1d). Importantly, DVSF-3 WT enhanced DENV infection of FcγR-bearing human K562 cells, whereas DVSF-3 LALA had no such ADE activity *in vitro* (Supplementary Fig. 2).

### Dengue DMAb delivery of DVSF-3 LALA protects against enhanced dengue disease in mice

**Figure 2:**
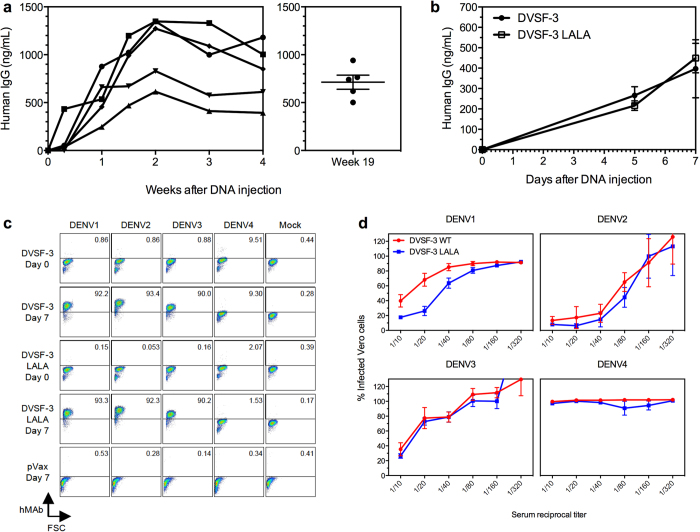
DMAb results in long-term expression of neutralizing DENV antibodies in mouse serum. (**a**) Total serum-detectable levels of human IgG was measured by ELISA after a single intramuscular injection of DNA plasmid encoding the anti-DENV human IgG antibody DVSF-1 into Foxn1/NuJ immunodeficient mice. Human IgG levels between weeks 0–4 (left; data displayed are mean of duplicate values each animal +/−SEM) and at week 19 (right; error bars display the mean of duplicate values from five animals +/− SEM). Each line (left) or dot (right) represents an individual mouse (n = 5 mice). (**b**) Total human IgG in serum was measured by ELISA after intramuscular injection of pDVSF-3 WT or pDVSF-3 LALA plasmids in 129/Sv mice (n = 4–5 mice per group, data displayed are the mean +/− SEM of each group’s animals and are representative of two independent experiments). (**c**) Vero cells were either uninfected (Mock) or infected by DENV1, 2, 3, or 4, then fixed, permeabilized, and stained with 129/Sv mouse serum taken at days 0 or 7 post-DNA injection of either pDVSF-3 WT, pDVSF-3 LALA or pVax empty vector (n = 5 mice per group, data representative of two independent experiments).(**d**) Neutralization was assessed by incubating DENV1, 2, 3, or 4 with serial dilutions of 129/Sv mouse serum taken at day 7 post-DNA injection of either pDVSF-3 WT or pDVSF-3 LALA (n = 5 mice per group) before addition to Vero cells. The percentage of infected cells is shown; error bars are the mean +/− SEM of each group’s animals).

In order to investigate antibody production kinetics *in vivo*, we determined the duration of DNA plasmid-encoded human IgG expression in nude mice, which would model antibody expression in an immune-accommodating host. The mice were injected intramuscularly with 100 μg of a DNA plasmid encoding another human IgG1 anti-DENV antibody, DVSF-1 WT (derived from DV82.11, a human IgG1 mAb that targets the DII fusion loop of the E protein and has been well characterized for its ability to neutralize DENV1–4^14^), followed immediately by EP. Human IgG concentrations in the serum were detectable within 5 days of injection, with peak levels of ~1000 ng/mL at two weeks post-injection (Fig. 2a, left panel). Duration of human IgG expression lasted at least 19 weeks (Fig. 2a, right panel), showcasing the sustained expression levels attainable with DNA plasmids. Given that the mouse DENV challenge model uses mice from the 129/Sv background, we sought to determine whether the antibody-encoding DNA plasmid constructs could produce serum-detectable levels of DVSF-3 WT or LALA in this background strain. Serum from 129/Sv mice receiving either pDVSF-3 WT or pDVSF-3 LALA showed comparable human IgG levels (Fig. 2b) and stained Vero cells infected with DENV1–3 (Fig. 2c). Additionally, whereas naïve sera was unable to neutralize virus in a neutralization assay, both WT and LALA-containing serum were capable of neutralizing DENV1-3 (Fig. 2d).

To assess whether mice expressing DNA plasmid-encoded anti-DENV neutralizing mAbs would be protected from DENV challenge, we employed the AG129 mouse model, which lacks type I and type II interferon (IFN) receptors and, upon DENV infection, recapitulates many aspects of human disease^30^,^31^. Importantly, these mice have been shown to exhibit ADE, with low doses of serotype-specific as well as cross-reactive antibodies both enhancing infection^30^. For these studies, mice were infected with the mouse-adapted DENV2 strain S221, which, in the presence of sub-neutralizing amounts of the anti-DENV mAb 2H2, causes antibody-enhanced severe disease and acute lethality (4–6 days post-infection) in AG129 mice at sublethal doses^30^.

**Figure 3:**
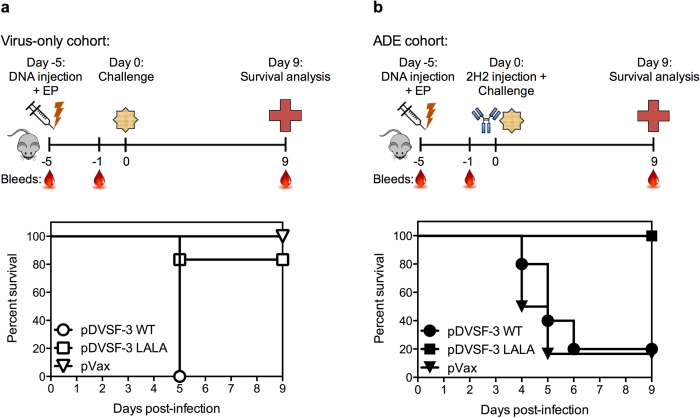
DMAb protects against virus-only and antibody-enhanced disease. (**a**) Virus-only challenge: AG129 mice received an intramuscular injection of either pDVSF-3 WT, pDVSF-3 LALA, or pVax empty vector five days prior to challenge with a sublethal dose of DENV2 S221 (n = 5–6 mice per group; p ≤ 0.0084 for comparison between pDVSF-3 LALA and pDVSF-3 WT).(**b**) Antibody-dependent enhancement challenge: AG129 mice received an intramuscular injection of either pDVSF-3 WT, pDVSF-3 LALA, or pVax empty vector five days prior to administration of an enhancing dose of the non-neutralizing anti-DENV mAb 2H2. Thirty minutes later, mice were challenged with a sublethal dose of DENV2 S221 (n = 5–6 mice per group; p ≤ 0.0072 for comparison between pDVSF-3 LALA and pDVSF-3 WT). A Kaplan-Meier survival curve is shown (a–b).

For challenge studies, AG129 mice were given a single intramuscular injection of pDVSF-3 WT or pDVSF-3 LALA followed immediately by EP. Negative controls received a single intramuscular injection of pVax1 empty vector followed by EP. Five days later, the mice were challenged with a sub-lethal dose (1 × 10^9^ GE) of DENV2 S221 in the presence (ADE) or absence (virus-only infection) of exogenous anti-DENV mAb 2H2. Mice in the pDVSF-3 WT, pDVSF-3 LALA, and pVax1 cohorts had mean human IgG concentrations of 750 ng/mL, 1139 ng/mL, and undetectable levels, respectively, one day before challenge (Supplementary Fig. 3; p ≤ 0.0930 for comparison between pDVSF-3 WT and pDVSF-3 LALA). Under virus-only infection conditions, we expect pDVSF-3 WT-treated mice to experience ADE and acute lethality, as immune complexes formed by DVSF-3 WT antibodies with DENV should lead to increased infection^14^. Conversely, we expect pVax1- and pDVSF-3 LALA-treated mice to survive, being unable to enhance disease. Indeed, five of six pDVSF-3 LALA-treated mice and all five pVax1 mice showed no lethal disease enhancement; all pDVSF-3 WT-treated mice succumbed to disease by day 5 (Fig. 3a; p ≤ 0.0084 for comparison between pDVSF-3 LALA and pDVSF-3 WT), demonstrating the non-enhancing functionality of pDVSF-3 LALA against virus-only infection. Under ADE conditions, we expect both pDVSF-3 WT- and pVax1-treated mice to experience acute lethality due to enhanced infection, whereas pDVSF-3 LALA-treated mice should be protected from severe disease. All five mice receiving pDVSF-3 LALA survived under ADE conditions, while those receiving either pDVSF-3 WT or pVax1 empty vector succumbed to acute, antibody-enhanced disease within 4–5 days (Fig. 3b; p ≤ 0.0072 for comparison between pDVSF-3 LALA and pDVSF-3 WT). Taken together, these data show that injection of pDVSF-3 LALA does not cause lethal enhancement after virus-only infection and protects against severe disease in ADE conditions, supporting the concept of muscle correctly processing and expressing functional antibodies from this platform.

### DMAb delivery of multiple antibodies increases human IgG concentration and breadth of viral coverage in mice

Given that DENV serotypes have been shown to escape neutralization^15^, it is likely that an antibody cocktail targeting multiple epitopes on the DENV virion would produce an ideal prophylactic strategy. DNA plasmids have been shown in numerous experiments to be delivered in multi-plasmid formulations^32^,^33^, suggesting that delivery of multiple antibody-encoding plasmids is feasible. To test this concept, we injected 129/Sv mice with pDVSF-3 WT (anti-DENV1–3) in one leg and pDVSF-1 WT (anti-DENV1–4) in the other. Mice injected with both plasmids had significantly higher serum human antibody levels at day 7 compared to mice receiving a single plasmid (Supplementary Fig. 4a; p ≤ 0.0088 for comparison between pDVSF-1 WT and pDVSF-1 + 3; p ≤ 0.0240 for comparison between pDVSF-3 WT and pDVSF-1 + 3). Furthermore, Sera from mice injected with both plasmids stained Vero cells infected with all four DENV serotypes (Supplementary Fig. 4b). We extended this analysis to their LALA variants, also including a plasmid encoding an additional anti-DENV4 neutralizing antibody, DVSF-2 (derived from DV22.3, a human IgG1 mAb that targets DI/DII of the E protein and has been well characterized for its ability to neutralize DENV4^14^). Mice injected with pDVSF-2 and pDVSF-3 were able to bind all four serotypes, and mice injected with all three antibodies had even greater binding against all four serotypes (Supplementary Figure 4c). These data suggest that DMAb can ultimately increase breadth of protection against infectious diseases.

## Discussion

The rising global health burden of dengue has created an enhanced urgency to develop a safe, inexpensive, and effective DENV vaccine that prevents both initial infection and ADE-induced severe disease. Here, a single intramuscular injection of a DNA plasmid encoding a modified human anti-DENV1-3 neutralizing antibody was capable of protecting mice against antibody-enhanced DENV disease without enhancing virus-only infection. The ability of DNA plasmids to encode protective Fc region-modified LALA antibodies is significant due to the inability of our immune system to produce ADE-preventing antibody variants upon DENV vaccination or natural infection. Since current vaccine candidates generate traditional antibodies, the potential of vaccines to inadvertently promote ADE, especially as vaccine protection wanes, is a serious concern. Delivering Fc region-modified LALA antibodies by DMAb that protect against both dengue fever and ADE-induced severe disease could be a unique alternative or addition to traditional vaccine approaches.

The protection conferred by neutralizing anti-DENV mAbs expressed by DMAb is very rapid, with complete survival in mice challenged less than a week after pDVSF-3 LALA administration – significantly more rapidly than vaccine-driven protection, which can take weeks to months to reach peak efficacy levels. The rapid induction of immunity may be advantageous to travelers, as well as the elderly or other populations who respond poorly to vaccines. Travelers to endemic regions frequently receive a number of intramuscular vaccinations prior to travel; as such, we could envision this approach being included alongside normal travel immunization regimens.

We demonstrated that plasmid-encoded DVSF-3 LALA serum levels of 1 ug/mL were protective against lethal enhanced dengue disease in mice. Previous work from Beltramello and colleagues showed that i.p. delivery of 1 to 5 ug of purified DV87.1 antibody 24 hours prior to DENV challenge was able to protect mice from lethal disease^14^; the protective levels observed in their study support our results here. Importantly, we demonstrated the delivery of multiple DENV DMAb plasmids in mice, which increased human IgG levels as well as the amount of serotypes targeted. This novel strategy could be used to increase the breadth of protective coverage against not just DENV, but also other infectious diseases. Furthermore, as monoclonal antibodies have proven to be efficacious against specific cancers or autoimmune disorders, employing DMAb to deliver monoclonal antibodies could be beneficial in such therapeutic antibody treatments and allow many such therapies to reach underserved populations. In summary, DMAb provides a rapid, novel delivery system for biologically relevant functional full-length monoclonal antibodies *in vivo*.

## Materials and Methods

### Cell lines, viruses, and reagents

Vero cells were kindly provided by Professor Robert Doms (Children’s Hospital of Philadelphia, Department of Pathology and Laboratory Medicine) and cultured in Medium 199 (Invitrogen) supplemented with 5% FBS and antibiotics (Invitrogen; 100 units/mL penicillin and 100 μg/mL streptomycin). K562 cells were purchased from ATCC and grown in Iscove’s Modified Dulbecco’s Medium (Invitrogen) supplemented with 10% FBS and antibiotics.

Dengue virus types 1 (TH-S-man; ATCC VR-1586), 2 (New Guinea C; ATCC VR-1584), 3 (Philippines/H87/1956; BEI Resources NR-80), and 4 (H241; BEI Resources NR-86) were amplified in Vero cells cultured at 37 ^o^C in Medium 199 supplemented with 2% FBS and antibiotics. FACS infectious units (IU)/mL were quantified by a flow cytometry-based viral titration assay^34^. Briefly, Vero cell monolayers were infected with serial dilutions of DENV for 24 hrs, after which infected cells positive for intracellular expression of the dengue E protein were enumerated by intracellular staining with the monoclonal antibody 4G2.

### Antibody Plasmid Construction

The DNA plasmids pDVSF-3 and pDVSF-3-LALA encode fully human IgG1 mAbs whose variable regions were derived from the anti-DENV1-3 human mAb DV87.1 [Genbank accession numbers: DV87.1 VH KC294015, DV87.1 VL KC294016]. Each transgene consisted of the heavy and light chain genes separated by a furin cleavage site coupled with a P2A self-processing sequence. The transgenes were codon and RNA optimized for expression in humans, synthesized by GenScript and cloned into modified pVax1 mammalian expression vectors (Invitrogen) under the control of the human cytomegalovirus immediate-early promoter.

### Western blots

For protein analysis, human antibodies were purified from supernatants of transfected cells with a Protein A antibody purification kit (Montage Antibody Purification Kit with PROSEP-A media, Millipore). Purified antibodies were separated in precast Bis-Tris gels (Invitrogen) under either reducing or nonreducing conditions. Proteins were transferred to Immobilon-FL PVDF transfer membranes (Millipore). Membranes were blocked for 1 hr in Odyssey Blocking Buffer (Li-Cor Biosciences), incubated with a goat anti-human IgG 680RD antibody (Li-Cor Biosciences), and washed. Protein bands were visualized on the Li-Cor Odyssey CLx.

### Animals, injection strategy, and challenge

Wild type 129/Sv mice were purchased from Charles River Laboratories. 129/Sv mice lacking both the IFN-α/β and γ receptors (AG129) were bred and housed at the La Jolla Institute for Allergy and Immunology (LIAI) Animal Facility. B6.Cg-Foxn1nu/J and C57BL/6 mice were purchased from The Jackson Laboratory. All animal housing and experimentation were approved by and conducted in accordance with the guidelines set by the NIH and the University of Pennsylvania Perelman School of Medicine or La Jolla Institute for Allergy and Immunology Institutional Animal Care and Use Committees.

Mice were administered a single 50 μL intramuscular (IM) injection of plasmid into the quadriceps followed by *in vivo* electroporation (EP) using a CELLECTRA adaptive constant current electroporation device (Inovio Pharmaceuticals Inc.) as previously described^29^. Serum samples were collected pre-injection and at various times after plasmid administration to determine human IgG antibody concentration, binding ability, and neutralization and enhancement capacity. All challenge experiments were performed five days after DNA administration in AG129 mice.

For DENV2 virus-only challenge experiments, 5 to 6 week-old AG129 mice were infected intravenously (via the tail vein) with 1 × 10^9^ genome equivalents (GE) of DENV2 strain S221 diluted in a total volume of 200 μL PBS with 10% FCS. For DENV2 enhanced disease challenge experiments, AG129 mice were administered 5 μg of the non-neutralizing anti-DENV mAb 2H2 intraperitoneally 30 minutes prior to infection with an intravenous 1 × 10^9^ GE dose of DENV2 strain S221.

### ELISA

For quantification of total human IgG, ELISA plates were coated with 1 μg/well of goat anti-human IgG-Fc fragment antibody (Bethyl) overnight at 4 ^o^C. Plates were blocked with 10% FBS in PBS for 1 hour at room temperature. After washing, samples were diluted in 1% FBS in PBS-T, added to the plate, and incubated for 1 hour at room temperature. Plates were washed, and HRP-conjugated goat anti-human kappa light chain (Bethyl) was added for 1 hr at room temperature. Sample was detected with SIGMA*FAST* OPD (Sigma-Aldrich). A standard curve was generated using purified human IgG/Kappa (Bethyl).

For determination of DENV E protein binding, ELISA plates were coated with 1 ug/mL of recombinant E protein from DENV1-4 (Fitzgerald Industries International) overnight at 4 ^o^C. Plates were blocked with 10% FBS in PBS for 1 hr at room temperature. After washing, samples were diluted in 1% FBS in PBS-T, added to the plate, and incubated for 1 hr at room temperature. Plates were washed, and HRP-conjugated goat anti-human IgG-Fc fragment antibody (Bethyl) was added for 1 hr at room temperature. Sample was detected with SIGMA*FAST* OPD (Sigma-Aldrich).

### Intracellular Staining

Vero cells were infected with DENV1 TH-Sman (ATCC VR-1586), DENV2 New Guinea C (ATCC VR-1584), DENV3 H87 (BEI Resources) or DENV4 H241 (BEI Resources) at an MOI of 0.01. After 5 days, cells were harvested, then were fixed, permeabilized, and washed with Cytofix/Cytoperm and Cytoperm/Cytowash (BD Biosciences). Cells were incubated with sample containing human anti-DENV antibodies diluted in Cytoperm/Cytowash for 1 hr on ice, washed, and then stained with goat anti-human IgG Fc FITC (Abcam) for 1 hr on ice. After washing, cells were analyzed on a LSRII (BD Biosciences).

### Flow cytometry DENV neutralization and enhancement assay

Vero cells were seeded in 100 μL of Medium 199 supplemented with 5% FBS and 1% PenStrep (Invitrogen) and plated at 5 × 10^3^ cells/well in flat-bottom 96-well plates. The next day, different dilutions of mAb-containing sample were incubated with 200 pfu/well of DENV for 1 hr at 37 ^o^C. The neutralization mixture was then added to Vero cells. After 3 days, cells were harvested, then were fixed, permeabilized, and washed with Cytofix/Cytoperm and Cytoperm/Cytowash (BD Biosciences). Cells were stained with 4G2 diluted in Cytoperm/Cytowash for 1 hr on ice, washed, and then stained with goat anti-mouse IgG Fc FITC (Abcam) for 1 hr on ice. After washing, cells were analyzed on a LSRII (BD Biosciences). To assess antibody-dependent enhancement, K562 cells were seeded in Iscove’s Modified Dulbecco’s Medium (IMDM) supplemented with 10% FBS at 5 × 10^3^ cells/well in flat-bottom 96-well plates and incubated with neutralization mixture formulated as above. After 3 days, cells were fixed, permeabilized, and washed as above. Cells were stained with 4G2, washed, and then stained with goat anti-mouse IgG Fc FITC before a final series of washes. Cells were then analyzed on a LSRII as above.

### Statistical analyses

All graphs were prepared using GraphPad Prism 6 (GraphPad Software). Survival data were expressed using Kaplan-Meier survival curves. A two-way ANOVA test was used to determine differences between multiple groups.

## Additional Information

**How to cite this article**: Flingai, S. *et al.* Protection against dengue disease by synthetic nucleic acid antibody prophylaxis/immunotherapy. *Sci. Rep.* **5**, 12616; doi: 10.1038/srep12616 (2015).

## Supplementary Material

Supplementary Information
