# Supplementary material for: Protection against dengue disease by synthetic nucleic acid antibody prophylaxis/immunotherapy
Source: Sci Rep. 2015 Jul 29;5:12616. doi: 10.1038/srep12616 (PMC4518257; doi:10.1038/srep12616)
Supplement: Supplementary Information [file srep12616-s1.pdf]

Supplementary Figures

<u>Manuscript title:</u>

Protection against dengue disease by synthetic nucleic acid antibody prophylaxis/immunotherapy

<u>Author List:</u>

Seleeke Flingai[1], Emily M. Plummer[2], Ami Patel[1], Sujan Shresta[2], Janess M. Mendoza[3], Kate E. Broderick[3], Niranjan Y. Sardesai[3], Kar Muthumani[1], David B. Weiner[1]*

[1]Department of Pathology and Laboratory Medicine, Perelman School of Medicine at the University of Pennsylvania, Philadelphia, PA 19104, USA.

[2]Division of Vaccine Discovery, La Jolla Institute for Allergy and Immunology, 9420 Athena Circle, La Jolla, CA 92037, USA

[3]Inovio Pharmaceuticals Inc., Plymouth Meeting, PA 19462, USA

*Corresponding author: David B. Weiner (dbweiner@mail.med.upenn.edu)

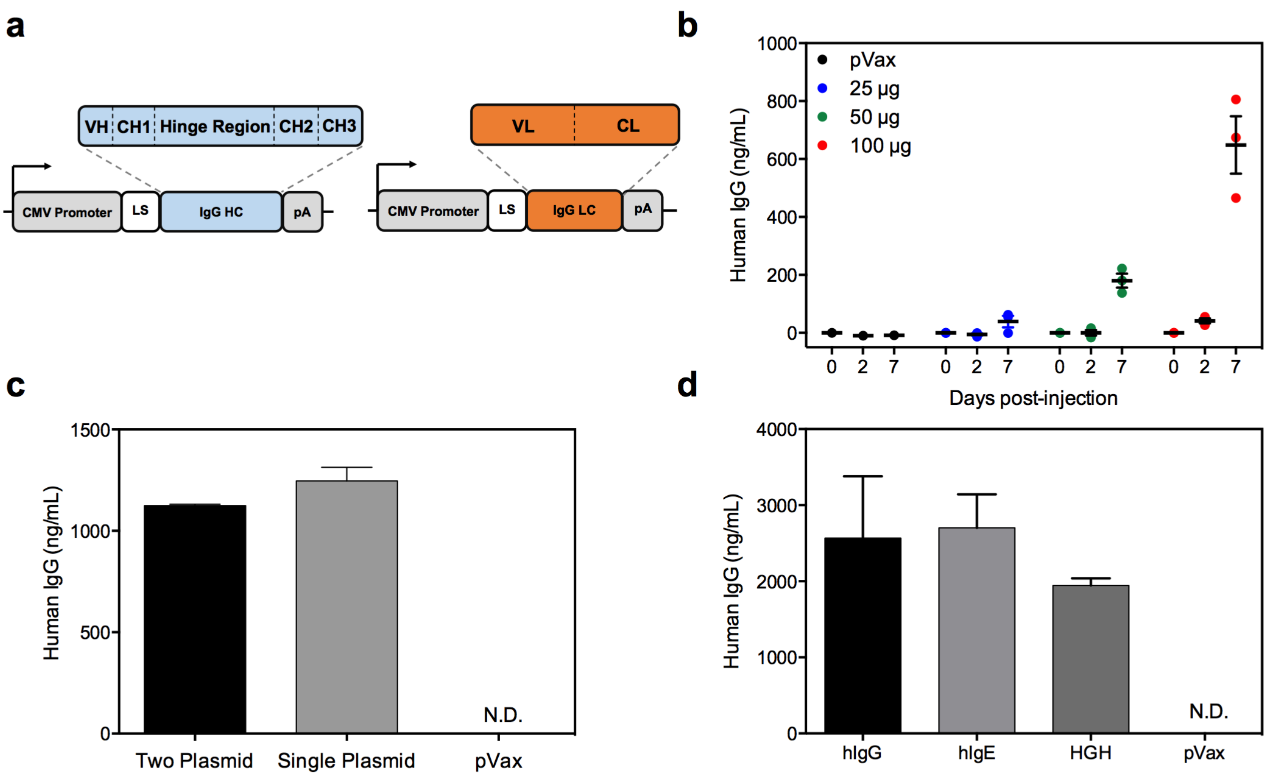

Supplementary Figure 1 Development and optimization of DMAb

(**a**) Schematic illustration of initial two-plasmid DMAb delivery system; human IgG antibody heavy and light chain sequences were expressed on separate plasmids co-delivered either *in vitro* or *in vivo* in a single formulation.
(**b**) Dosage study for two-plasmid DMAb delivery of human IgG in C57B/6 mice measured by ELISA; DNA amounts indicate total DNA injected intramuscularly followed by EP (100 µg = 50 µg IgG heavy chain DNA + 50 µg IgG light chain DNA; n = 3 mice per group).
(**c**) Comparison of human IgG levels *in vitro* by ELISA from two-plasmid DMAb or single plasmid DMAb (samples run in duplicate; data representative of two independent experiments).
(**d**) Comparison of human IgG levels *in vitro* by ELISA from single plasmid DMAb incorporating different human signal sequences fused to the IgG heavy and light chain genes (samples run in duplicate; data representative of three independent experiments).

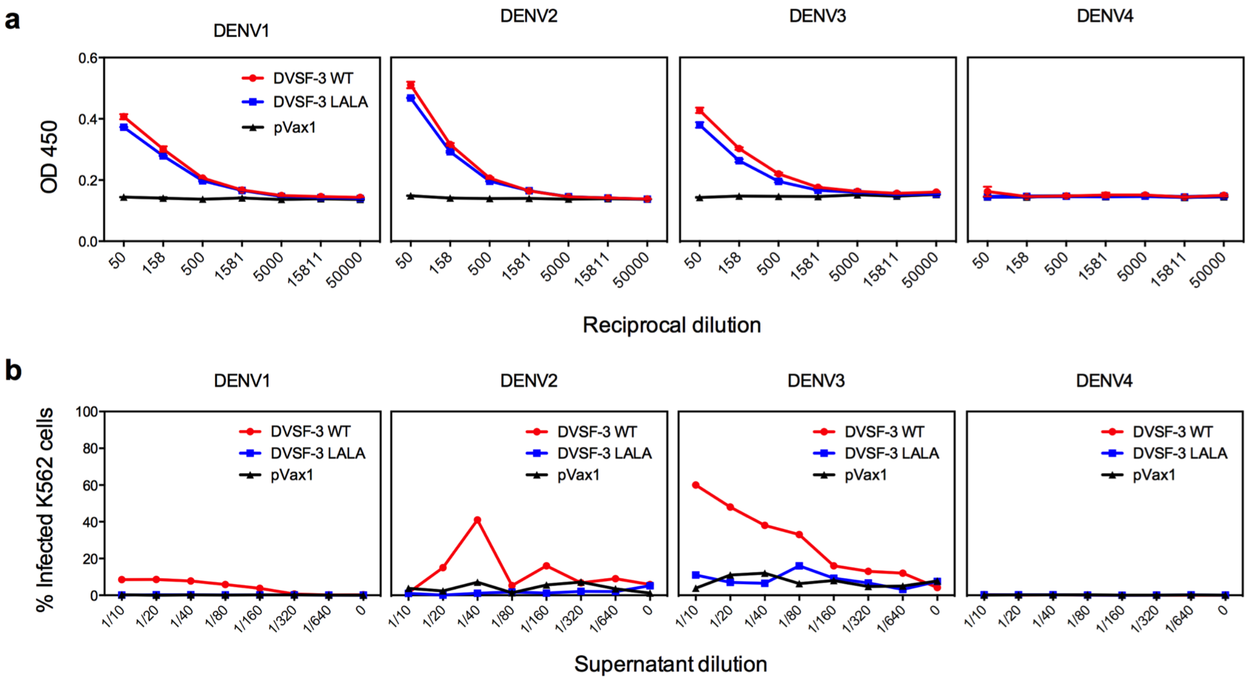

Supplementary Figure 2 *In vitro* functional analysis of pDVSF-3 WT and LALA-encoded antibodies

(**a**) ELISA binding analysis of human IgG in supernatants of pDVSF-3 WT- or LALA-transfected 293T cells against purified recombinant DENV E proteins (samples run in duplicate; data representative of two independent experiments).
(**b**) Antibody-dependent enhancement were assessed by incubating DENV1, 2, 3, or 4 with serial dilutions of supernatants of pDVSF-3 WT- or LALA-transfected 293T cells before addition to K562 cells. The percentage of infected cells is shown (samples run in duplicate; data representative of two independent experiments).

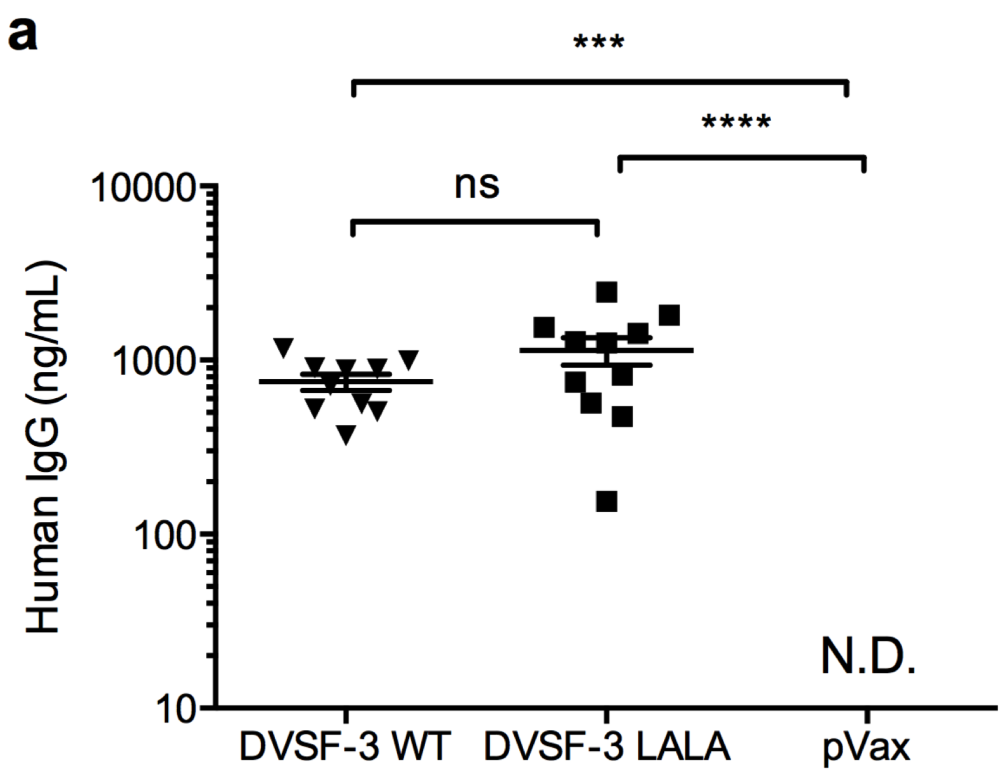

Supplementary Figure 3 Pre-challenge levels of anti-DENV human IgG levels in AG129 mice after DMAb delivery

(**a**) Total human IgG of DVSF-3 WT or DVSF-3 LALA in serum was measured by ELISA 4 days after DNA intramuscular injection (one day before DENV2 challenge) and EP of respective plasmids in AG129 mice ($n = 10$-11 mice per group; $p \leq 0.0930$ for comparison between pDVSF-3 WT and pDVSF-3 LALA; $p \leq 0.0005$ for comparison between pDVSF-3 WT and pVax; $p \leq 0.0001$ for comparison between pDVSF-3 LALA and pVax).

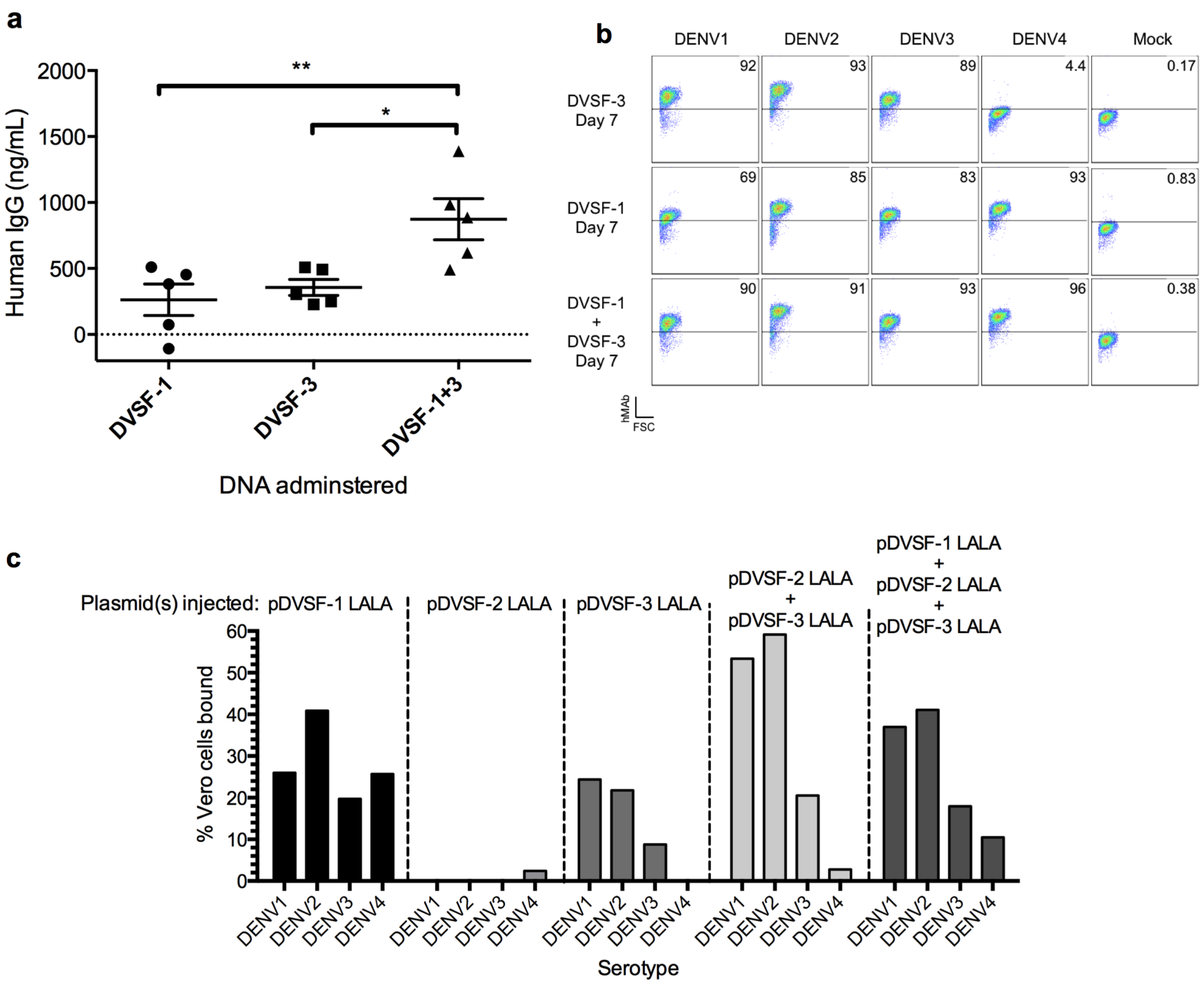

Supplementary Figure 4 Delivery of multiple DENV antibody-encoding plasmids in mice produces increased DENV1-4 antisera

(**a**) Total human IgG of DVSF-3 WT, DVSF-1 WT, or DVSF-3 WT and DVSF-1 WT in serum was measured by ELISA 7 days after DNA intramuscular injection and EP of 100 μg of respective plasmids in 129/Sv mice (n = 5 mice per group; $p \leq 0.0088$ for comparison between pDVSF-1 WT and pDVSF-1+3; error bars represent standard error of the mean; $p \leq 0.0240$ for comparison between pDVSF-3 WT and pDVSF-1+3). (**b**) Vero cells were either uninfected (Mock) or infected by DENV1, 2, 3, or 4, then fixed, permeabilized, and stained with 129/Sv mouse serum taken at days 0 or 7 post-DNA injection of either pDVSF-3 WT (100 μg), pDVSF-1 WT (100 μg), or pDVSF-3 WT and pDVSF-1 WT (100 μg of each in separate legs) (n = 5 mice per group, representative of two independent experiments). (**c**) Bar graph representing the breadth of 129/Sv mouse serum staining of Vero cells infected with DENV1, 2, 3, or 4 after injection of single (pDVSF-1 LALA, pDVSF-2 LALA, or pDVSF-3 LALA) or multiple (pDVSF-2 LALA + pDVSF-3 LALA, or pDVSF-1 LALA + pDVSF-2 LALA + pDVSF-3 LALA) plasmids (n = 4 mice per group; data representative all mice from each respective group)
